# Cytotoxic Autophagy in Cancer Therapy

**DOI:** 10.3390/ijms150610034

**Published:** 2014-06-05

**Authors:** Khushboo Sharma, Ngoc Le, Moureq Alotaibi, David A. Gewirtz

**Affiliations:** Department of Pharmacology and Toxicology, Massey Cancer Center, Virginia Commonwealth University, Richmond, VA 23298, USA; E-Mails: sharmak3@mymail.vcu.edu (K.S.); lenb@vcu.edu (N.L.); alotaibimr@vcu.edu (M.A.)

**Keywords:** autophagy, apoptosis, radiation, chemotherapy

## Abstract

Autophagy is a process of cellular self-digestion, whereby the cell degrades subcellular materials in order to generate energy and metabolic precursors in order to prolong survival, classically under conditions of nutrient deprivation. Autophagy can also involve the degradation of damaged or aged organelles, and misfolded or damaged proteins to eliminate these components that might otherwise be deleterious to cellular survival. Consequently, autophagy has generally been considered a prosurvival response. Many, if not most chemotherapeutic drugs and radiation also promote autophagy, which is generally considered a cytoprotective response, in that its inhibition frequently promotes apoptotic cells death. Furthermore, it has been shown that conventional chemotherapeutic drugs and radiation alone rarely induce a form of autophagy that leads to cell death. However, there are multiple examples in the literature where newer chemotherapeutic agents, drug combinations or drugs in combination with radiation promote autophagic cell death. This review will describe autophagic cell death induced in breast tumor cells, lung cancer cells as well as glioblastoma, demonstrating that it cannot be concluded that stress induced autophagy is, of necessity, cytoprotective in function.

## 1. Introduction

Programed cell death (PCD) is a terminal path for removal of abnormal or damaged cells, such as those subjected to genotoxic damage, in order to maintain homeostasis. At least four major types of programed cell death have been identified including apoptosis and autophagy [1,2], mitotic catastrophe and necrosis [3,4]. Thus, cell death can occur by different mechanisms and the phenotypic changes that accompany cell death can vary depending on the stimulus and cellular settings. 

Autophagy has generally been considered to be a survival or cytoprotective response during growth factor withdrawal or under stressful conditions such as gamma-radiation, toxic stimuli, and chemotherapy [1,2,5,6,7]. In certain cellular settings, however, autophagy can serve as a cell death mechanism [8]. Thus, it is important to distinguish between cytoprotective autophagy and the cellular settings in which autophagy can cause cell death, *i.e.*, where autophagy is cytotoxic [2,5,8]. (Although we have identified two additional forms/functions of autophagy, specifically nonprotective and cytostatic autophagy [9], this review will focus solely on the cytotoxic form). Shen and Codogno [8] have proposed three criteria to define cell death by autophagy: (i) cell death occurrence is independent of apoptosis; (ii) there is an increase in autophagic flux; (iii) genetic or pharmacologic suppression of autophagy prevents cell death. However, we believe that the first two criteria may not always apply and that the third criterion is the critical one to define cytotoxic autophagy [10].

Studies by Shen *et al.* [11] in which the autophagy regulatory gene, *Atg7*, was knocked down demonstrated that most conventional cancer chemotherapeutic drugs fail to promote cytotoxic autophagy. However, this review will present studies relating to cytotoxic autophagy in three tumor model systems, breast cancer, lung cancer and glioma/glioblastoma demonstrating that it is generally the newer drug classes and unconventional drug combinations or drug-radiation combinations that promote autophagic cell death.

Recent studies in different tumor cell lines have demonstrated that tumor resistance to anticancer therapies such as radiation therapy and chemotherapy is often associated with up-regulation of autophagy [12,13]. However, a growing body of evidence implicates a paradoxical role of autophagy following anticancer treatments, with autophagy induction also mediating the antitumor action of various therapeutics [9]. Thus, understanding the pathophysiology of the disease together with the functional relevance of autophagy within the tumor is critical to efforts to circumvent resistance and enhance the effects of anticancer therapies for cancer patients.

## 2. Autophagic Cell Death in Breast Tumor Cells

Breast cancer is the second leading cause of cancer-related death in women in the United States [14]. It is one of the most common malignancies where accumulation of abnormal cells is possibly due to disordered autophagy regulation and imbalanced cell proliferation and apoptosis [15]. 

*Beclin-1* and *Atg5* are two genes that have been shown to be required for the promotion of autophagy [16,17], and consequently interfering with *Beclin-1* and/or *Atg5* expression should reduce autophagy and protect against autophagic cell death. Two independent pathways that can theoretically induce autophagic cell death include canonical and non-canonical autophagy [15,16,17,18]. In the canonical autophagy pathway, Beclin-1 is essential for the initiation of autophagy through its interaction with the class III phosphatidylinositol-3-kinase Vps34 [18]. Interaction of Beclin-1 with the anti-apoptotic Bcl-2-family proteins Bcl-2 and Bcl-xL inhibits Beclin-1 function while the expression of *Beclin-1* mutants that are unable to associate with Bcl-2 leads to a high level of autophagy and cell death.

Studies conducted by Akar *et al.* [17] have shown that when Beclin-1 function is left unchecked by silencing of Bcl-2, excessive levels of autophagy induce cell death in MCF-7 breast tumor cells exposed to doxorubicin. In these studies, autophagy was monitored based on increased levels of acridine orange staining, expression of LC3-II (a protein associated with the membrane of the autophagosome) and Beclin-1 expression while the absence of apoptosis induction was based on low or insignificant levels of Annexin V staining and PARP (poly(ADP-ribose)polymerase) cleavage. Furthermore, silencing of Atg5 inhibited doxorubicin induced autophagic cell death in Bcl-2 silenced cells. This is interesting from the standpoint that silencing of the anti-apoptotic protein, Bcl-2, might otherwise be expected to be permissive for apoptosis. 

In contrast to canonical autophagy, non-canonical autophagy can contribute to cell death through a process that does not use the entire machinery of Atg proteins (such as Beclin-1) to form autophagosome [16]. Scarlatti *et al.* [16] reported that resveratrol, a polyphenol found in grapes and peanuts, induced Beclin-1 independent cytotoxic autophagy in MCF-7 cells. It was shown that resveratrol inhibited MCF-7 cell proliferation and promoted cell death (based on Trypan blue exclusion and clonogenic survival assays). In addition, resveratrol induced typical biomarkers of autophagy such as an increase in LC3-I and accumulation of LC3-II in the presence of the lysosomal inhibitors E64 and pepstatin A. However, silencing of Beclin-1 or hVPS34 neither abrogated resveratrol-induced autophagosome formation (measured by LC3-II accumulation and punctated GFP-LC3) nor resveratrol-induced cell death. Moreover, resveratrol-induced cell death was not reversed by treatment with z-VAD, a caspase inhibitor. Instead, introduction of Atg7 siRNA significantly suppressed autophagosome formation and reduced cell death induced by treatment with resveratrol in MCF-7 cells. Taken together with the work of Akar *et al.* [17] these findings indicate that there are at least two independent mechanisms that can induce autophagic cell death, the canonical autophagy pathway and the non-canonical pathway acting independently of the tumor suppressor Beclin-1.

Autophagy serving as a type-II programed cell death pathway appears even more complex when multiple levels of dialog between autophagy and apoptosis are considered [1,19]. In some circumstances, both autophagy and apoptosis are required in parallel pathways to contribute to cell death [2]. For instance, Shrivastava *et al.* [20] showed in a recent study that the cannabinoid, cannabidiol (CBD), could suppress the survival of both estrogen receptor-positive and estrogen receptor-negative breast tumor cells by inducing both apoptosis and autophagy. 

In eukaryotic cells, endoplasmic reticulum (ER) stress plays an important role in the induction of autophagy in response to multiple cellular stressors [21]. In this context, CBD treatment of MDA-MB231 cells showed a significant increase in the phosphorylation of EIF2α, which is a putative marker of ER stress. Furthermore, CBD mediated autophagy by inhibiting the AKT/mTOR/4EBP1 signaling pathway (a pathway that is frequently activated in human cancers and modulates cancer metastasis, cancer cell proliferation, and acquired drug resistance), as observed by a decrease in the phosphorylated forms of these proteins. Concurrent with the inhibition of AKT/mTOR signaling, CBD elevated cleaved PARP and LC3-II levels, markers for the induction of apoptosis and autophagy, respectively, suggesting that CBD may induce cell death in a complex interplay between apoptosis and autophagy in MDA-MB231 breast tumor cells. Inhibition of autophagy using Bafilomycin, which inhibits the acidification of lysosomes, late endosomes and autolysosomes as well as the fusion of autophagosomes with lysosomes, suppressed autophagic cell death and favored apoptotic cell death as shown by an increase in Annexin V positive cells and PARP cleavage levels. These data suggest that blocking CBD-induced autophagy promotes a compensatory increase in apoptosis as an alternative means of programed cell death.

In addition to the parallel promotion of autophagy and apoptosis described above, autophagy can serve as a part of the apoptotic program [2,19] as well as a backup cell death mechanism after caspase inhibition [22]. In this context, studies conducted by Di *et al.* [23] demonstrated that suppression of apoptosis in MDA-MB231 and MCF-7/E6 breast tumor cells (cells lacking functional *p53*) treated with doxorubicin using the broad spectrum caspase inhibitor, z-VAD-Fmk, resulted in substantial induction of autophagy. In this study, autophagy was assessed based on acridine orange and monodansylcadaverine dye staining 2 days after initiating exposure to 1 µM doxorubicin in cells pretreated with z-VAD-Fmk. It is critical to emphasize that the induction of autophagy did not alter the sensitivity of z-VAD-Fmk treated cells to doxorubicin as measured by clonogenic survival assays, indicating that autophagy contributed to cell death in response to doxorubicin. These findings indicate that when stress-induced apoptosis is blocked, autophagy can provide an alternative cell death mechanism. 

It is further of importance to mention the potential intraconversion between different functions of autophagy. For instance, studies conducted by Wilson *et al.* [24] and Bristol *et al.* [25] demonstrated that there could be a switch between cytoprotective and cytotoxic autophagy in breast tumor cells treated with radiation either alone or in combination with vitamin D (1,25-dihydroxy vitamin D_3_; 1,25-D_3_) or a vitamin D analog. While a number of studies, including our own, have demonstrated that radiation alone induces cytoprotective autophagy [24,25,26,27,28], treatment of ZR-75-1 or MCF-7 breast tumor cells with 1,25-D_3_ and irradiation promoted autophagic cell death as a mode of radiation sensitization [24,25]. Evidence for sensitization was based on a decrease in clonogenicity and a reduction in cell viability compared to radiation alone. The conclusion that autophagic cell death was the mode of sensitization was based on the increase in acridine orange staining, the formation of punctate RFP-LC3, as well as an increase in autophagic vesicle formation quantified by FACS (fluorescence-activated cell sorting) analysis. Minimal apoptosis was detected with the combination treatment as measured by the TUNEL (terminal deoxynucleotidyl transferase dUTP nick end-labeling) assay and DAPI (4',6-diamidino-2-phenylindole) staining, Annexin/PI staining, western blots analysis for both PARP cleavage and Caspase-3 cleavage, and FACS analysis for a sub-G1 population. Most critically, pharmacologic or genetic inhibition of 1,25-D_3_-radiation-induced autophagy (by bafilomycin A1 or chloroquine, and knockdown of Atg5 or Atg7) resulted in reduced radiation sensitivity where cell viability was restored to levels similar that observed with irradiation alone. On the other hand, inhibition of irradiation-induced cytoprotective autophagy resulted in a decrease in cell viability [24,25]. These findings indicate that breast tumor cells can be sensitized to radiation through the promotion of autophagic cell death.

**Figure 1:**
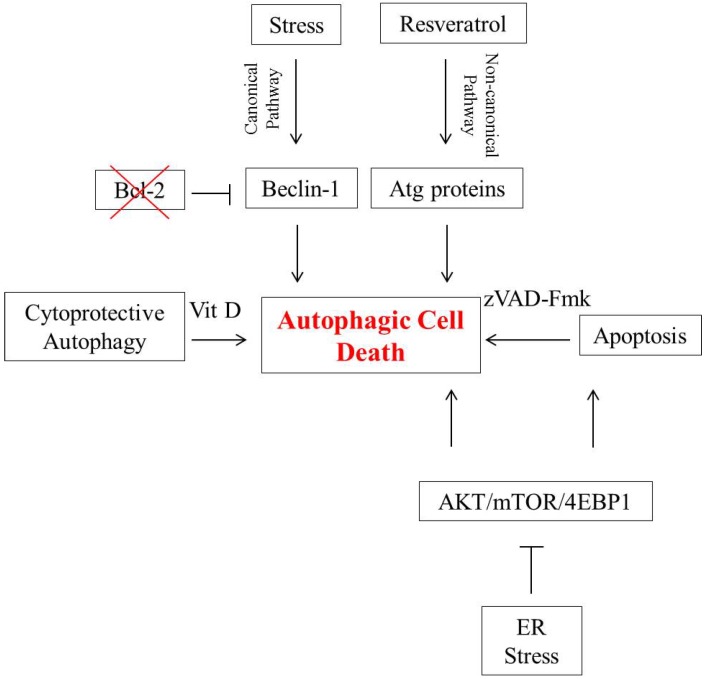
Autophagic cell death in breast tumor cells. In the canonical pathway, Beclin-1 is essential for the initiation of autophagy. Bcl-2 binds to Beclin-1 and interferes with its capacity to promote autophagy; conversely, silencing of Bcl-2 can lead to autophagic cell death. Autophagy induced in the non-canonical pathway is independent of Beclin-1 function. Resveratrol is one agent that induces non-canonical autophagic cell death in breast tumor cells. Endoplasmic reticulum (ER) stress mediates autophagy by inhibiting the AKT/mTOR/4EBP1 signaling pathway and can induce both apoptosis and autophagic cell death. Autophagic cell death can serve as a backup mechanism in cells where apoptosis is inhibited, by agents such as the caspase inhibitor, z-VAD-Fmk. Intraconversion between cytoprotective autophagy and autophagic cell death can be observed in MCF-7 and ZR-75-1 breast tumor cells when radiation treatment is combined with vitamin D or a vitamin D analog.

The studies described above are summarized in Figure 1.

## 3. Autophagic Cell Death in Lung Cancer Cells

Lung cancer has a relatively poor prognosis with a very low median survival rate and is one of the leading causes of death in United States [29]. Surgery is usually the standard treatment for NSCLC but the majority of NSCLC patients are not eligible for surgical resection [30,31,32]. Unfortunately, currently available therapies such as radiation and chemotherapy are usually only palliative [30,31,32]. 

As is the case with breast cancer, while there are many examples of autophagy having cytoprotective functions in lung cancer cells [28,33,34], there is also a significant body of evidence for the cytotoxic function of autophagy [35,36].

The phytochemical, Anacardic Acid (AA), is known to have anti-cancer, anti-inflammatory, anti-oxidant, anti-obesity and anti-bacterial effects. In studies of the role of AA in ER stress induced autophagy, Seong *et al.* [37] demonstrated an increase in autophagy related proteins such as Atg5 and Beclin-1 and an increase in formation of autophagosomes by electron microscopy. The endoplasmic reticulum is involved in the process of initiating autophagy and, at the same time, autophagy is required to maintain ER homeostasis [37]. The GRP78/BiP protein appears to be required for ER integrity and stress induced autophagy in mammalian cells [38]. Studies by Seong *et al.* [37] further showed that AA inhibits the PERK-eIF2 signaling pathway and increases levels of GRP78/BiP, ATF6 and IRE1. This inhibition of PERK-eIF2 signaling further promoted an increase in accumulation of nascent proteins in the ER associated with ER stress and in autophagy, leading to cell death. DAPK (death-associated protein kinase 1), a calcium/calmodulin-regulated serine/threonine protein kinase, is known to be a mediator of autophagic cell death induced in response to ER stress generated by AA [39]. DAPK has been shown to cause phosphorylation of Beclin1, leading to its dissociation from Bcl-xL [40]. Beclin 1 further activated PI3K (phosphoinositide 3-kinase), leading to formation of autophagosomes and hence an increase in autophagy as shown by TEM (transmission electron Microscope). However, these studies did not directly determine whether autophagy was the mode of cell death through e.g., monitoring the impact of autophagic inhibitors on sensitivity to AA.

Autophagy has also been shown to increase in cancer cell models where apoptosis, the type I programed cell death pathway, has been blocked or resistance has developed. In studies by Kim *et al.* [41] which addressed the potential utility of inhibiting both apoptosis and mTOR signaling to improve the efficacy of radiation therapy, the H460 NSCLC cell line was shown to be more sensitive to radiation treatment in the presence of a caspase (apoptosis) inhibitor, Z-DEVD; sensitivity was further increased when radiation was used in combination with RAD001, which promotes autophagy through the inhibition of mTOR. *In vivo* studies using H460 xenografted tumors confirmed the *in vitro* findings and showed that the combination treatment induced a significant delay in tumor growth compared to radiation alone. These studies suggest that sensitization can occur even if levels of apoptosis are significantly reduced. These findings were confirmed by evidence for increased levels of autophagy (by GFP-LC3 induction) with the combination treatment of radiation, Z-DEVD and RAD001 both *in vivo* and *in vitro*. An increase in LC3II protein levels for the combination treatment also supported the increase in autophagy. Furthermore, when autophagy related genes, *Atg5* and *Beclin-1*, were knocked down, radiosensitization was reversed, implicating autophagy as being responsible for the radiosensitization and reduced cell proliferation as shown by a clonogenic survival assay.

This group published similar data supporting the cytotoxic functions of autophagy using *Bak*/*Bax*^−/−^ lung tumor cells in combination with the mTOR inhibitor, RAD001 [42]. The Bcl-2 family plays major roles in the process of apoptosis and the Bax and Bak members of this family regulate caspase mediated cell death. *Bak*/*Bax*^−/−^ cells were defective in undergoing apoptosis but were more radiosensitive than wild type cells, ostensibly through an increase in autophagy and pro autophagic proteins. Inhibition of autophagy attenuated the radio-sensitization of *Bak*/*Bax*^−/−^ cells, whereas overexpression of autophagy related genes *Atg5* and *Beclin-1* made wild type cells radiosensitive, implicating autophagy as the mode of radio-sensitization and cell death.

CUB ((C1r/C1s, urchin embryonic growth factor, BMP1) domain containing protein 1, CDCP1) is a type I transmembrane protein, which has been reported in several malignancies such as lung cancers and breast tumors, mostly associated with poor prognosis and disease progression [43,44]. Specifically, phosphorylation of CDCP1 is found to regulate cell migration, invasion and anoikis resistance of cancer cells [45,46]. Anoikis is a form of cell death triggered by loss of interaction with the extracellular matrix [47]. In studies conducted by Miyazawa *et al.* [48], autophagy was shown to be involved in anoikis of lung cancer cells caused by suppression of CDCP1. Cell death induced by knocking down CPCD1 in A549 (NSCLC) cells under suspension conditions was caspase 3 (a marker for apoptosis) independent. z-VAD-Fmk, a general inhibitor of caspase, did not have any effect on the cell death caused by knock down of CDCP1, confirming that cell death being induced in lung cancer cells by inhibition of CDCP1 signaling is caspase independent. LC3II protein levels were shown to increase for lung cancer cells with knock down of CDCP1 levels and the increased autophagy was confirmed by GFP-LC3 foci formation. Furthermore, the increase in cell death in A549 cells treated with CDCP1 siRNA was rescued/reversed by treatment with 3-methyl adenine (3-MA), which inhibits the initiation of autophagy.

Pemetrexed, which is a folate anti-metabolite, is approved for the treatment of NSCLC and has been shown to regulate autophagy [49,50]. In studies by Bareford *et al.* [50] that were designed to understand the role of autophagy in response to pemetrexed and in combination therapy with sorafenib, a multikinase inhibitor, the drugs acted synergistically to enhance tumor growth inhibition *in vivo* by promoting autophagy. Genetic silencing of genes such as *Beclin-1* and *Atg5* demonstrated that sensitization effects could be reversed when autophagy was blocked or inhibited in lung tumor cells. It was further determined that inhibition of the mTOR/p70s6k signaling pathway, which results in the promotion of autophagy, played a central role in cell killing by the combination treatment. 

Studies conducted by Khan *et al.* [51] have also implicated autophagy as being involved in the process of cell death by iron oxide nanoparticles. Cell death was found to be reduced in lung cancer cells upon treatment with 3-MA. Inhibition of the classical AMPK-mTOR-AKT pathway was implicated in the induction of autophagy by iron oxide nanoparticles in NSCLC cells. Experiments using the free radical scavenger, *N*-acetyl cysteine also indicated the involvement of ROS (reactive oxygen species) in hyper activation of autophagy and cell death. Another factor that was shown to be playing a role in the promotion of autophagic cell death was mitochondrial damage, which was assessed by measuring the levels of mitochondrial membrane potential (MMP). Mitochondrial depolarization leads to changes in cellular ATP levels, which when sensed by the energy sensor, AMPK, leads to its phosphorylation and autophagy induction via suppression of the mammalian target of rapamycin (m-TOR) [52]. 

Kim *et al.* [53] showed that ionizing radiation led to a significant increase in autophagy and that this autophagy compromised survival of NSCLC HCC827 cells. Quite unexpectedly, since radiation induced autophagy is quite frequently cytoprotective, a Beclin-1 silenced cell line was used to show that autophagy contributed to radiosensitization by an increase in cell survival and a decrease in LC3II levels in the siBeclin cell line compared to siControl cells. Knockdown of PTEN, an established tumor suppressor gene, which is found to be inactivated in a number of cases of non-small cell lung cancer increased resistance and reduced sensitivity of NSCLC cells to radiation as well as gefitinib, which is a commonly used to treat lung cancer [53]. Cytotoxic autophagy was induced in response to radiation in NSCLC cells using an mTOR inhibitor to overcome PTEN knockdown induced radioresistance. 

The tumor suppressor gene, *p53*, which is uniformly activated in response to DNA damage by radiation, can induce autophagy through the activation of autophagy inducing genes, particularly ULK1 and ULK2. This activation of ULK1 and ULK2 lead to an elevation in autophagy levels in response to DNA damage and contributes to cell death [54]. A possible mechanism by which activated mTOR inhibits cytotoxic autophagy is by preventing ULK1 activation [55]. Thus, loss of PTEN followed by an increase in mTOR activity could lead to an increase in clonogenic survival. Therefore, the studies by Kim *et al.* [53] suggest that inhibiting mTOR signaling could be an effective strategy to increase radiosensitivity.

**Figure 2:**
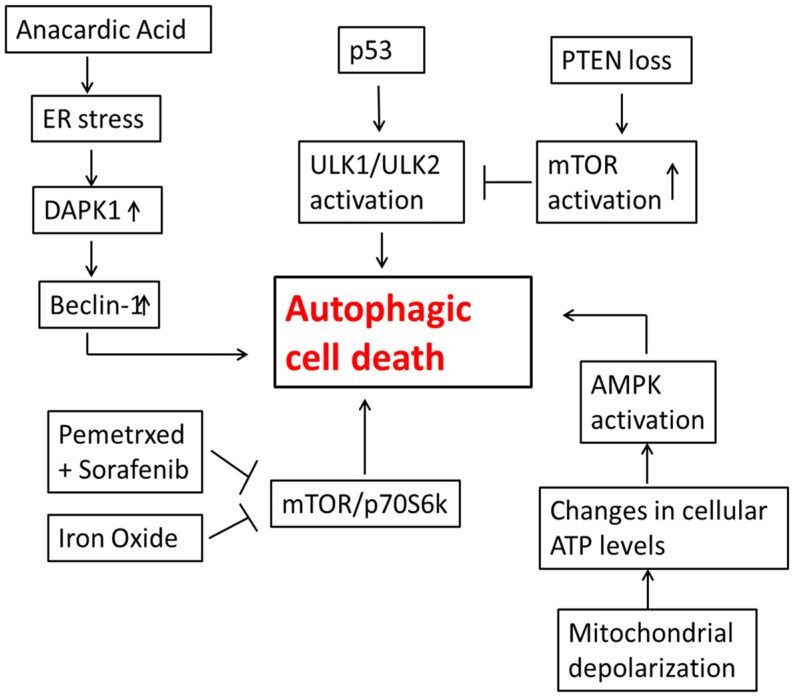
Autophagic cell death in lung tumor cells. The combination of pemetrexed and sorafenib as well as iron oxide alone can result in the inhibition of mTOR, leading to autophagic cell death. Alterations in cellular ATP levels due to mitochondrial depolarization have been shown to cause activation of AMPK, an established initiator of autophagy. Activation of p53 up regulates the ULK1/ULK2 pathway; not shown is that this may occur downstream of AMPK activation. Anacardic acid increases ER (endoplasmic reticulum) stress, which can further lead to an increase in Beclin-1 levels, a key regulator of autophagy. Loss of PTEN promotes activation of mTOR, which leads to inhibition of ULK1/ULK2 and interference with autophagy induction.

Another commonly used chemotherapeutic drug to treat lung cancer is Cisplatin. However, its use is limited due to acquired resistance from prolonged treatment. Studies conducted by Sirichanchuen *et al.* [56] showed a reduction in autophagy to be the underlying mechanism for this acquired resistance. Using two H460 cells lines, one sensitive to cisplatin (H460) and one rendered resistant to cisplatin by exposure to gradually increasing concentrations of drug (H460/cis), these investigators established that a reduction in autophagy levels led to a decrease in cisplatin-mediated cell death, supporting a cytotoxic function of autophagy. Furthermore, resistance could be reversed, making H460/cis cells more sensitive to treatment, by inducing autophagy using TFP (Trifluoperazine). Conversely, the chemosensitizing effects of TFP could be reversed using the autophagy inhibitor, 3-MA. Mechanistically, long-term cisplatin treatment has been shown to cause over expression of Bcl-2, which as indicated above, acts as a negative regulator of autophagy via its interaction with Beclin-1 [57]. Consistently in this study Bcl-2 was shown to be higher in the H460/cis cell line implicating its role in reduced autophagy in cisplatin-resistant cells (H460-cis) compared to parental NSCLC cells (H460).

The studies described above are summarized in Figure 2.

## 4. Autophagic Cell Death in Glioblastoma

Malignant glioblastomas are the most common form of brain cancer. Late stages of brain cancer are associated with poor prognosis and the average survival rate of patients diagnosed with malignant glioma generally does not exceed two years [58,59]. While treatment of glioblastoma includes radiotherapy and chemotherapy, generally, glioblastomas (GBMs) are resistant to the current therapeutics due to the hyperactivation of the PI3K-Akt-mTOR survival pathway by excessive stimulation via growth factor receptors and Ras [60]. Most GBMs carry mutations on the PTEN gene, whose function is to inhibit the continuous activation of PI3K/Akt [60,61]. As the apoptotic pathway is also frequently inactivated due to genetic mutations [58,62], GBMs are considered to be relatively resistant to cytotoxic insults [63,64].

Since mutations in GBMs often inactivate the apoptotic pathway, malignant glioblastomas are likely to be more sensitive to autophagic cell death as an alternative response to therapeutics [58,65]. In studies on rapamycin-sensitive malignant U87-MG and T98G glioma cells, it was found that autophagy was induced but not apoptosis; in contrast, as might have been expected, rapamycin-resistant cells failed to show autophagy [66]. Akt and PI3K inhibitors enhanced sensitivity of these cells to rapamycin by increasing the extent of autophagy [66] and appeared to synergistically promote cell death. 

The function of autophagy is not always clear. For instance, studies have shown that inhibition of PI3K and mTOR has a cytostatic, but not a cytotoxic effect, both *in vitro* and *in vivo* [67]. Because autophagy is negatively regulated by the PI3K-Akt-mTOR pathway, interference with this pathway promotes autophagy. While pharmacologic and genetic inhibition of autophagy along with inhibition of PI3K-Akt-mTOR pathway resulted in apoptosis, this does not necessarily mean that autophagy was functionally cytoprotective since the cells were actually arrested, presumably through the promotion of autophagy [9].

In response to radiation, the PI3K-Akt-mTOR pathway is activated and frequently mediates resistance [68,69,70,71]. Several studies have shown that inhibition of PI3K/Akt/mTOR signaling sensitizes tumor cells to different chemotherapeutic strategies [72,73]. Since glioblastoma cells appear to be dependent on this pathway for proliferation [60,61], targeting this pathway is likely to prove to be a useful therapeutic strategy. Fujiwara *et al.* [74] concluded that the use of the Akt inhibitor, 1L-6-hydroxymethyl-chiro-inositol 2(*R*)-2-*O*-methyl-3-*O*-octadecylcarbonate, radio-sensitized the radioresistant malignant glioma U87-MG cell line by enhancing autophagy but not apoptosis. 

Studies using adenoviruses, a relatively new and promising approach for glioma treatment, indicated that glioma cells as well as glioma stem cells undergo autophagic cell death [75,76]. Conditionally replicating adenoviruses (CRAds) have the ability to multiply and specifically lyse cancer cells. Ito *et al.* [75] showed that a conditionally replicating adenovirus alone can have a cell killing effect in both U373-MG and U87-MG cells. The cytotoxic effect of CRAds was associated with induction of autophagy but not apoptosis as shown in Hoechst staining. Furthermore, inhibition of autophagy by 3-methyl adenine suppressed the cytotoxicity of CRAds in U373-MG and U87-MG cells, indicating that CRAds enhances autophagic cell death. 

Another study that confirmed the potential impact of oncolytic adenoviruses in brain tumors involved four brain tumor stem cell lines from surgical glioblastoma specimens that were infected with an oncolytic adenovirus, Delta-24-RGD [76]. Consistent with the previous report, infecting glioblastoma cells with the adenovirus induced autophagy, as demonstrated by the accumulation of acidic vacuoles and clear induction of Atg5 both *in vivo* and *in vitro*. The anti-tumor activity of Δ-24-RGD was clearly evident based on an increase in the mean survival time of the treated mice from 38.5 to 66.3 days. However, it was not directly established that autophagy was responsible for tumor cell killing. 

The malignant glioma M059K cell line shows high resistance to radiation, probably due to the ability to rapidly repair radiation-induced DNA damage [77,78,79]. Generally, autophagy induced in response to low doses of radiation in the M059K cells seems to be cytoprotective and cells retain their proliferative recovery 48 h later. A deficiency in the DNA-PK catalytic subunit in the counterpart M059J cells results in autophagic cell death in response to low doses of radiation, with apoptosis in a relatively small fraction of the cell population [80,81,82]. In support of this finding, antisense oligonucleotides against DNA-PKcs in other malignant glioblastoma cell lines, U373-MG and T98G, radio-sensitized these cells through enhancement of autophagy [81]. Furthermore, glioma initiating cells (GIC) transfected with shRNA targeting DNA-PKcs, an important component of the holoenzyme DNA-PK, confirmed the promotion of autophagy without evidence of apoptosis [82]. The reason for the absence of DNA-PKcs promoting cytotoxic autophagy upon radiation is still unclear, but may be mediated through the targets phosphorylated by DNA-PKcs, specifically two Ku subunits [83], XRCC4 [84], *p53* [85], MDM2 [86], and c-Abl [87]. Each one of the downstream targets has a different role during DNA-PK activation but Ku and XRCC4 phosphorylation seems to be linked to DNA repair whereas *p53*, MDM2 and c-Abl control apoptosis mediated through DNA-PK. 

Glioblastomas have demonstrated sensitivity to Temozolomide, a compound that forms O^6^-methylguanine in DNA, resulting in mispairing with thymine [88,89,90]. Several studies have indicated that Temozolomide induces G2/M arrest and that cells subsequently die through autophagy, but not apoptosis [91,92]. Furthermore, in support of this conclusion, O^6^-benzylguanine, an inhibitor of O^6^-methylguanine methyltransferase, augmented the effect of Temozolomide through an increase in the promotion of autophagy, with no evidence of apoptosis. Interference with autophagy via 3-methyladenine suppressed the sensitivity of malignant glioblastoma cells to the Temozolomide, indicating that sensitivity of GBMs to Temozolomide is mediated through autophgy. 

Finally, a recent study by Palumbo *et al.* [93] clearly showed how different forms of autophagy may be involved in the response to treatment in glioblastoma either by radiation alone or in combination with temozolomide. Using two cell lines that differ in their sensitivity to radiation, T98G and U373MG cells, at low doses of radiation ~2 Gy, T98G cells showed high sensitivity to radiation compared to U373MG cells, associated with the promotion of autophagy with minimal evidence for apoptosis. Furthermore, pretreatment of both cell lines with rapamycin, a known autophagy inducer, sensitized the resistant cells to radiation. Of critical importance, inhibition of autophagy in T98G cells by silencing ATG-7 and Beclin1 significantly attenuated the cytotoxicity of low doses of radiation as well as the combination of radiation and Temozolomide. 

Taken together, these findings suggest that induction of autophagy in glioblastoma cell lines could present a promising strategy to promote cell death and achieve improved therapeutic outcomes. 

**Figure 3:**
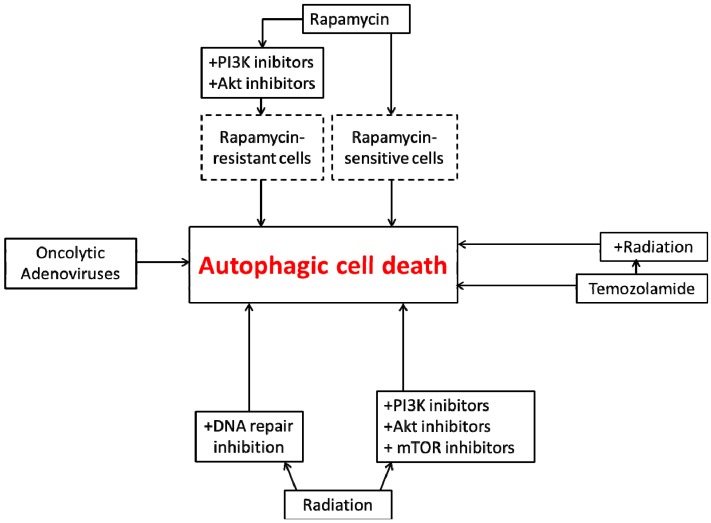
Autophagic cell death in glioblastoma cells. Rapamycin, an mTOR inhibitor, can directly induce autophagy and autophagic cell death in drug- sensitive glioblastoma cells. In rapamycin-resistant cells, inhibition of PI3K and Akt is required in order to induce autophagic cell death. Both radiation and temozolomide can induce autophagy individually, but their combination is more effective in promoting autophagic cell death. DNA repair inhibitors, PI3K inhibitors, Akt inhibitors, and mTOR inhibitors can confer radiation sensitivity and promote autophagic cell death. Oncolytic viruses also promote autophagic cell death.

The studies described above are summarized in Figure 3.

In summary, multiple studies in at least three different tumor model systems indicate that autophagy frequently is associated with antiproliferative and cytotoxic functions; consequently, it cannot be safely assumed that when a therapeutic modality promotes autophagy, that autophagy will serve a cytoprotective function and/or confer resistance to the treatment modality. Since there is no a priori indication whether autophagy is cytoprotective or cytotoxic, this must be determined through assessment of the impact of pharmacological and genetic inhibition of autophagy on tumor cell sensitivity to the autophagy-inducing stress.

One very important caveat should be mentioned here. Virtually all of the studies described in this review were performed in cell culture and there is little evidence in the literature that induction of autophagy can actually lead to tumor cell death in animal models of cancer. 

## 5. Conclusions

Although the literature relating to autophagy induction in tumor cells tends to largely focus on the cytoprotective function of this response, there is extensive evidence in a variety of experimental tumor models that autophagy can also have a cytotoxic function. However, this generally does not occur in response to conventional chemotherapeutic drugs or radiation alone but rather in response to unconventional agents or drug/drug and drug/radiation combination treatments. Nevertheless, the fact that autophagy can be cytotoxic in function indicates the importance of determining the mode of autophagy that is being induced by a particular treatment modality before autophagy inhibition can be considered as a potential clinical strategy for enhancing the response of malignancies to therapy.
